# How Do ROS Induce NETosis? Oxidative DNA Damage, DNA Repair, and Chromatin Decondensation

**DOI:** 10.3390/biom14101307

**Published:** 2024-10-16

**Authors:** Dhia Azzouz, Nades Palaniyar

**Affiliations:** 1 Translational Medicine, Peter Gilgan Centre for Research and Learning, The Hospital for Sick Children, Toronto, ON M5G 0A4, Canada; 2 Department of Laboratory Medicine and Pathobiology, University of Toronto, Toronto, ON M5S 1A8, Canada; 3 Institute of Medical Sciences, Faculty of Medicine, University of Toronto, Toronto, ON M5S 1A8, Canada

**Keywords:** reactive oxygen species (ROS), molecular mechanisms, mitochondria, transcriptional firing, apoptosis during NET formation (ApoNETosis), innate immune proteins, NET formation, DNA repair, DNA damage

## Abstract

Neutrophil extracellular traps (NETs) are intricate, DNA-based, web-like structures adorned with cytotoxic proteins. They play a crucial role in antimicrobial defense but are also implicated in autoimmune diseases and tissue injury. The process of NET formation, known as NETosis, is a regulated cell death mechanism that involves the release of these structures and is unique to neutrophils. NETosis is heavily dependent on the production of reactive oxygen species (ROS), which can be generated either through NADPH oxidase (NOX) or mitochondrial pathways, leading to NOX-dependent or NOX-independent NETosis, respectively. Recent research has revealed an intricate interplay between ROS production, DNA repair, and NET formation in different contexts. UV radiation can trigger a combined process of NETosis and apoptosis, known as apoNETosis, driven by mitochondrial ROS and DNA repair. Similarly, in calcium ionophore-induced NETosis, both ROS and DNA repair are key components, but only play a partial role. In the case of bacterial infections, the early stages of DNA repair are pivotal. Interestingly, in serum-free conditions, spontaneous NETosis occurs through NOX-derived ROS, with early-stage DNA repair inhibition halting the process, while late-stage inhibition increases it. The intricate balance between DNA repair processes and ROS production appears to be a critical factor in regulating NET formation, with different pathways being activated depending on the nature of the stimulus. These findings not only deepen our understanding of the mechanisms behind NETosis but also suggest potential therapeutic targets for conditions where NETs contribute to disease pathology.

## 1. Introduction

The immune system is a complex network of cells and molecules working together to defend the body against infections and diseases [1]. It is composed of two systems: the innate immune system and the adaptive immune system [2]. The innate immune system provides a rapid, non-specific defense against pathogens, while the adaptive immune system offers a slower, highly specific response that includes memory for long-term protection [3,4,5]. Among the key players are neutrophils, macrophages, T cells, and B cells, each with distinct roles in immunity, inflammation, autoimmune diseases, cancer, and wound healing [6].

Neutrophils are the first responders to infection, rapidly arriving at the site to engulf pathogens through phagocytosis [7]. They release enzymes and antimicrobial proteins to kill bacteria [8,9]. In autoimmune diseases, overactive neutrophils can attack healthy tissues, contributing to conditions like rheumatoid arthritis [10,11]. They can also release neutrophil extracellular traps (NETs) which can result in the production of autoantibodies, as will be discussed below [12,13]. In cancer, neutrophils can have dual roles: they can attack tumor cells but also promote tumor growth and metastasis by creating an inflammatory environment [14,15]. During wound healing, neutrophils clear pathogens and debris from the wound site, initiating the healing process and signaling other immune cells to aid in repair [16].

Macrophages are versatile cells that play a crucial role in both immunity and inflammation [17]. They engulf and digest pathogens, dead cells, and debris, and present antigens (pieces of pathogens) to T cells, initiating the adaptive immune response [18,19]. In autoimmune diseases, macrophages can perpetuate inflammation and tissue damage by continually presenting self-antigens to T cells [20]. In cancer, tumor-associated macrophages (TAMs) often support tumor growth and suppress anti-tumor immunity [21]. During wound healing, macrophages transition from a pro-inflammatory state (M1) to an anti-inflammatory state (M2), promoting tissue repair and regeneration by secreting growth factors and cytokines [22].

T cells are essential for adaptive immunity. Helper T cells (Th cells) coordinate the immune response by activating other immune cells, including B cells and macrophages, while cytotoxic T cells (Tc cells) directly kill infected or cancerous cells [23,24]. T cells secrete cytokines that modulate inflammation: Th1 cells promote inflammation to combat intracellular pathogens, whereas Th2 cells support responses against extracellular parasites and help regulate allergic reactions [25,26,27]. Regulatory T cells (Tregs) suppress excessive immune responses, preventing autoimmunity and maintaining immune balance [28]. In autoimmune diseases, T cells can mistakenly attack the body’s own tissues [29]. In cancer, T cells can recognize and kill tumor cells, but tumors often develop mechanisms to evade T cell detection [30]. During wound healing, T cells regulate inflammation and assist in the repair process by secreting growth factors [31].

B cells are pivotal in producing antibodies that specifically target pathogens [32]. When activated by antigens and helper T cells, B cells differentiate into plasma cells that secrete large amounts of antibodies [33]. These antibodies neutralize pathogens and mark them for destruction by other immune cells. B cells influence inflammation through antibody production and cytokine secretion [34]. Some B cells secrete pro-inflammatory cytokines, while others have anti-inflammatory effects, helping to balance the immune response [35]. In autoimmune diseases, B cells can produce autoantibodies that target the body’s own tissues, leading to conditions such as lupus [36,37]. In cancer, B cells can produce antibodies that target tumor cells, but they can also contribute to a pro-tumor environment [38]. During wound healing, B cells help modulate the inflammatory response and aid in tissue repair through the production of antibodies and cytokines [39].

Together, these cells orchestrate a finely tuned immune response. Neutrophils provide rapid defense and initiate acute inflammation. Macrophages engage in pathogen clearance, antigen presentation, and regulation of inflammation. T cells coordinate adaptive immunity and directly attack infected cells, while B cells produce antibodies and help regulate the inflammatory response. This coordinated effort ensures effective defense against infections, maintains tissue health, and prevents excessive inflammation, while also playing critical roles in autoimmune diseases, cancer, and wound healing.

## 2. NETosis

While all the aforementioned cell types are equally important to human health and disease, this review will focus on neutrophils, specifically the role of reactive oxygen species in NETosis. Neutrophil extracellular traps (NETs) are web-like structures composed of DNA, histones, and antimicrobial proteins released by neutrophils [8,40]. These structures play a crucial role in the immune system’s defense against infections. When neutrophils encounter pathogens, they can release NETs to trap and neutralize these invaders, preventing their spread and facilitating their destruction by other immune cells [41]. NETs are especially effective against bacteria, fungi, and parasites, providing a powerful mechanism to contain infections and support the immune response [42].

However, the role of NETs in disease is complex, as their dysregulation can contribute to various pathological conditions, including autoimmune diseases [12,43]. In autoimmune diseases, the components of NETs, such as DNA and histones, can become autoantigens [44,45]. When these normally hidden cellular components are exposed, the immune system may mistakenly recognize them as foreign and produce autoantibodies against them [46]. This can lead to a sustained immune response against the body’s own tissues, contributing to diseases like systemic lupus erythematosus (SLE) and rheumatoid arthritis [47,48].

Additionally, NETs can play a role in cancer and chronic inflammatory conditions. In cancer, NETs can have dual effects: they can trap and kill tumor cells, but they can also create a pro-inflammatory environment that supports tumor growth and metastasis [49]. Chronic inflammation induced by persistent NET formation can damage tissues and contribute to the progression of diseases such as atherosclerosis and chronic obstructive pulmonary disease (COPD) [50,51].

In the context of wound healing, NETs can be beneficial by preventing infection and promoting the clearance of debris. However, excessive NET formation can impair the healing process by sustaining inflammation and causing tissue damage [52,53]. Therefore, while NETs are an essential component of the immune response, their regulation is critical to prevent their contribution to disease pathology. Balancing the beneficial and harmful effects of NETs remains a significant challenge in managing various inflammatory and autoimmune diseases.

NETosis is a form of cell death specific to neutrophils. The Nomenclature Committee of Cell Death has defined NETosis as “A ROS-dependent modality of RCD [regulated cell death] restricted to cells of hematopoietic derivation and associated with NET extrusion” [54]. NETs are made of DNA decorated with cytotoxic proteins. NETosis refers to cases of NET formation that end in cell death, since not all cases of NET formation end in cell death [55].

NETosis, which is independent of apoptosis and necrosis, was first observed by Takei et al. in 1996 [8]. The research group induced the novel type of cell death using the activator phorbol 12-myristate 13-acetate (PMA), which is still used as a canonical NOX-dependent NETosis inducer [8]. Takei et al. also uncovered that the novel form of cell death requires ROS production, as determined using the antioxidants thiourea, dimethylthiourea, pyrrolidinethiocarbamate, and N-acetyl-L-cysteine [8].

This process was further characterized by Brinkmann et al., who uncovered the ability of NETs to kill bacteria as well as the involvement of NETs in acute inflammation, such as during spontaneous human appendicitis [9]. Since its discovery, several types of NETosis have been characterized [56].

### 2.1. NETosis Mechanisms

The best-characterized types of NETosis are NADPH oxidase 2 (NOX)-dependent and NOX-independent NETosis. However, the distinction between these types is not as clear-cut as it might seem. Research has revealed a range of variants that occur under different conditions, influenced by various stimuli and environmental factors. These discoveries highlight the complexity of NETosis and demonstrate that neutrophils can respond to a broad spectrum of triggers in diverse ways. The nuances in how NETosis operates reflect the adaptability of the immune system in different scenarios.

#### 2.1.1. NOX-Dependent NETosis

NOX-dependent NETosis is particularly well-studied and involves a series of well-defined steps. This process is typically triggered by stimuli such as phorbol 12-myristate 13-acetate (PMA), lipopolysaccharides (LPSs), and various bacteria like *Pseudomonas aeruginosa* [8,9]. PMA activates protein kinase C (PKC), which then leads to the activation of NADPH oxidase [57,58]. LPSs, a component of bacterial cell walls, and certain pathogenic bacteria can directly stimulate neutrophils to undergo NETosis [59].

Once neutrophils are activated, the NADPH oxidase enzyme complex is assembled from several subunits, including NOX2 itself, gp91^phox^, p22^phox^, p47^phox^, p40^phox^, and p67^phox^ [60]. This complex then produces reactive oxygen species (ROS) from molecular oxygen. The ROS generated by NOX are crucial for initiating and driving the NETosis process. This is followed by the activation of mitogen-activated protein kinases (MAPKs), such as extracellular signal-regulated kinase (ERK), p38 MAPK, and c-Jun N-terminal kinase (JNK) [59,61,62]. These MAPKs play a significant role in modulating various cellular processes, including gene expression. This is followed by transcriptional firing and chromatin decondensation. The final step is the release of NETs, composed of decondensed chromatin and antimicrobial proteins, into the extracellular space [56,59,63].

#### 2.1.2. NOX-Independent NETosis

NOX-independent NETosis is another distinct form of NET formation, characterized by its unique triggering mechanisms and cellular processes. This type of NETosis can be initiated by calcium ionophores such as A23187 and ionomycin, as well as by uric acid and ultraviolet (UV) light exposure [56,64,65,66]. These stimuli induce NETosis through different pathways compared to NOX-dependent NETosis.

In NOX-independent NETosis, the primary driver of NET formation is the increased generation of ROS by mitochondria rather than by NADPH oxidase [65]. This mitochondrial ROS production is crucial for the subsequent steps of NET formation. The following step is the activation of mitogen-activated protein kinase p38 (MAPK p38), which plays a key role in mediating cellular responses to stress and inflammation. Activated MAPK p38 then influences transcriptional machinery within the neutrophil, ramping up transcriptional firing [63,65].

A critical aspect of NOX-independent NETosis triggered by calcium ionophores is the increase in intracellular calcium levels. This rise in calcium activates peptidylarginine deiminase 4 (PAD4), an enzyme responsible for the citrullination of histones [67]. Citrullination is a post-translational modification where arginine residues in histones are converted to citrulline at promoter regions [65,68]. As a result, histones become less tightly bound to DNA, allowing for the extension of chromatin fibers and the formation of NETs.

Moreover, while NETosis has traditionally been viewed as a form of cell death, research has identified a non-lytic variant known as vital NETosis. Unlike conventional NETosis, which typically involves cell death and the release of NETs, vital NETosis allows neutrophils to release NETs while remaining viable [69]. This form of NETosis represents a more nuanced understanding of how neutrophils contribute to immune responses without necessarily undergoing complete cell death.

### 2.2. Ultraviolet Light, a Recently Uncovered Inducer of NETosis

UV light is a well-known inducer of intrinsic apoptosis, primarily through the production of mitochondrial ROS [70]. Given this common factor with apoptosis, we investigated UV’s ability to induce NETosis at higher doses, which should result in elevated levels of mitochondrial ROS. It was found that high doses of UV irradiation indeed induce NETosis, whereas low doses trigger apoptosis. Specifically, UV-induced NETosis occurs rapidly and follows the typical pattern of NOX-independent NETosis. As UV doses increase, there is greater activation of the apoptotic pathway, particularly caspase 3, along with increased DNA decondensation and NET formation. Notably, this occurs without the classical DNA condensation seen in apoptosis. To distinguish between apoptosis [71] and NETosis [72], immunolabeling was employed to detect cleaved caspase 3 and myeloperoxidase (MPO). MPO, which is located in neutrophil granules [73], migrates to the nucleus during NETosis [72], marking decondensed chromatin and being released as part of the NETs. These observations confirmed that higher UV doses enhance NETosis.

Mitochondrial ROS production and p38 MAPK activation were identified as key factors in this increased NETosis. Inhibitor studies using a NOX inhibitor, diphenyleneiodonium chloride, demonstrated that UV-induced NETosis operates independently of NOX [56]. Instead, UV irradiation led to a dose-dependent increase in mitochondrial ROS. Blocking mitochondrial ROS with the uncoupler 2,4-dinitrophenol (DNP) [65] and the ROS scavenger MitoTempo [74] inhibited UV-induced NETosis. This demonstrates that mitochondrial ROS are crucial for UV-induced NETosis, similar to its role in A23187-induced NOX-independent NETosis [65].

Unlike calcium ionophore-induced NETosis, this process does not involve histone citrullination [65,75]. Calcium ionophore-induced NETosis involves highly activated PAD4 that is translocated into the nucleus to citrullinate histones [76]. Calcium ionophores increase intracellular calcium levels, which may explain the citrullination difference between calcium ionophore- and UV-mediated NOX-independent NETosis [77].

However, transcriptional activity remains essential for DNA decondensation. Further investigation revealed that UV-induced NETosis represents a distinct form of cell death, differing from apoptosis, necrosis, and necroptosis. This new form, termed apoNETosis, features both apoptotic and NETotic processes, with NETotic events predominating [64,78].

Since our initial discovery of UV-induced NETosis, several subsequent studies by others have provided additional insights. While we found that UV-C induces NOX-independent NETosis, other types of UV light may generate different forms of ROS, as indicated by other studies [79].

One study specifically examined UVA-induced NETosis and found that it relies on NOX activation but does not involve histone citrullination [80]. In contrast, another group investigated UVB-induced NETosis in vivo and reported the presence of citrullinated histones in the tissue [81]. However, it is important to note that in vivo data can be influenced by various factors, including hepoxilin and cytokines, which could induce histone citrullination independently of UVB exposure. Thus, the observed citrullination of histones may not solely be attributed to UVB radiation. A similar phenomenon is observed with LPS stimulation. In vitro, LPSs do not induce citrullinated histones, whereas in vivo studies show histone citrullination following LPS treatment [59,82]. Furthermore, a study examining UVA- and UVB-induced NETosis in vitro did not observe histone citrullination [83]. Instead, the authors concluded that the process was NOX-dependent based on their use of the NOX inhibitor, DPI. However, it is worth noting that the concentration of DPI used in this study was high. Another study has shown that red, blue, and long-wave UV light can also induce NETosis in neutrophils [80,84]. These findings suggest that flavins can capture electrons excited by both the visible and invisible light spectra to generate ROS. Despite this, the significant suppression of NETosis by mitochondrial ROS inhibitors indicates that mitochondrial ROS are the primary contributors to UV-induced NETosis. Regardless, whether NOX-dependent or NOX-independent, the essential factor remains that some form of ROS are necessary for UV-induced NETosis to occur.

### 2.3. Alternative Types of NETosis

There are some alternative types of NETosis that have been uncovered. These types may involve distinct stimuli, mechanisms, or characteristics compared to classical NETosis. Yousefi et al. demonstrated that viable neutrophils, when primed with GM-CSF and stimulated by C5a or TLR4 ligand LPSs, can produce NETs. These NETs formed by living neutrophils contain mitochondrial DNA but not nuclear DNA. The process is dependent on ROS and does not lead to neutrophil death. Additionally, the stimulated neutrophils exhibit increased survival compared to untreated neutrophils that do not generate NETs [85].

Clark et al. found that platelet TLR4 detects TLR4 ligands (e.g., LPSs) in the blood, leading to platelet binding to neutrophils [86]. This triggers neutrophil activation and the formation of NETs. Plasma from septic patients also induces platelet–neutrophil interactions and NET production. NETs effectively trap bacteria in blood vessels, particularly in the liver and lungs.

A study by Pilsczek et al. revealed a rapid and distinct mechanism of NET formation in response to *Staphylococcus aureus* ATCC 25923 [87]. It does not involve neutrophil death or membrane rupture. Instead, the nucleus undergoes changes, and vesicles filled with DNA are released intact and rupture to expel the compartmentalized chromatin. This process occurs within 5–60 min and is NOX-independent. The resulting NETs exhibit proteolytic activity and effectively kill *S. aureus* ATCC 25923. Panton–Valentine leukocidin was identified as the primary inducer. Similarly, Yipp et al. uncovered a novel form of vital NETosis wherein neutrophils became anuclear cytoplasts capable of phagocytosing Staphylococcus [69].

Chen et al. uncovered that the human neutrophil Fcγ receptor FcγRIIIB internalizes soluble immune complexes (ICs) and results in the formation of NETs in tissues [88]. Interestingly, NETosis induced by ICs does not rely on NADPH oxidase, myeloperoxidase, or neutrophil elastase. Studies have also shown that various microcrystals, such as monosodium urate crystals associated with gout, can induce the formation of NETs [89]. Microcrystals are insoluble crystals of different compositions and shapes, known to trigger an inflammatory response and release of NETs.

A recent study uncovered that both apoptosis and NETosis can occur within the same neutrophils, contrary to previous beliefs. This phenomenon, observed when neutrophils were treated with ultraviolet light, has been termed “apoNETosis”. Notably, apoNETosis is independent of NOX activity and involves mitochondrial ROS [64,78,90,91,92,93,94,95,96,97].

LL37, a human antimicrobial peptide that stimulates ROS production in neutrophils [98,99], plays a significant role in NETosis [100]. LL37 enhances the formation of NETs by promoting the release of DNA and antimicrobial proteins from neutrophils, thereby boosting the immune response [101]. This peptide not only helps in trapping and neutralizing microbes but also modulates inflammation, making it a crucial factor in both innate immunity and the regulation of NETosis [102,103].

## 3. Reactive Oxygen Species (ROS)

A common element found in NOX-dependent and -independent NETosis is the production of ROS. The primary source of ROS responsible for PMA-induced NETosis was determined to be NOX-derived by Fuchs et al. [104]. This was determined using the NOX inhibitor diphenylene iodonium (DPI) as well as by the observation that neutrophils obtained from chronic granulomatous disease patients (who carry mutations in NADPH oxidase) lack the ability to form and release NETs [104]. The involvement of ROS in NETosis was once again in the spotlight when, in 2015, Douda et al. uncovered the presence and necessity of mitochondrial ROS production in NOX-independent NETosis induced by calcium ionophores [56]. Mitochondrial ROS inhibition using 2,4-Dinitrophenol (DNP) and the mitochondrial ROS scavenger MitoTEMPO reduced calcium ionophore-induced NETosis [56].

In the case of NOX-dependent NETosis, NOX produces O_2_^−^, which is catalyzed by superoxide dismutase to H_2_O_2_, which is then catalyzed by myeloperoxidase to HOCl. H_2_O_2_ can also be changed to OH^.^ radicals. O_2_^−^, HOCl, and OH^.^ radicals oxidize many macromolecules within the cell, including DNA. In the case of NOX-independent NETosis, the mitochondria are the source of the ROS production required for NETosis. Mitochondrial ROS production occurs in the electron transport chain (ETC), where Complex II acts as the primary source [105]. It has also been shown that the 2-oxoacid dehydrogenase and the pyruvate dehydrogenase complexes can produce a significant amount of mitochondrial ROS [106]. The ROS produced by the ETC are in the form of superoxide [105]. Superoxide can oxidize many macromolecules within the neutrophil, including DNA.

ROS can have a wide range of effects within cells, including the oxidation of critical macromolecules, such as DNA, lipids, and proteins. Among these, DNA is particularly significant, especially in the context of NETs, which are primarily composed of DNA along with antimicrobial proteins. Given the central role of DNA in NETs, this review will focus on the impact of ROS on DNA and the subsequent repair processes.

ROS induce oxidative damage to DNA by causing chemical modifications to its bases. This oxidative damage can lead to a variety of alterations, such as base modifications and strand breaks, which can disrupt the normal structure and function of DNA. If these modifications are not repaired, they can result in mutations, potentially leading to genomic instability and contributing to various diseases.

To counteract this damage, cells possess sophisticated DNA repair machinery designed to recognize and correct oxidative modifications [107]. This repair machinery includes several pathways, such as base excision repair and nucleotide excision repair. Each pathway involves a series of enzymes and proteins that identify damaged sites, remove oxidized bases or fragments, and restore DNA to its original state.

## 4. DNA Repair Pathways

The genomes of cells are under consistent threat of damage from many endogenous and exogenous sources, such as ROS and UV. These sources can result in lesions in the form of incorrect and modified bases (such as 7,8-dihydro-8-oxoguanine (8-oxoG) by ROS), adduct formation (such as pyrimidine adducts by UV), single-strand breaks (SSBs), and double-strand breaks (DSBs). Such lesions can lead to impaired transcription, DNA replication stalling, and incorporation of incorrect bases. Hence, many cell types have evolved to repair damage to their DNA [108]. UV light has previously been reported to induce ROS in neutrophils [109].

Nucleotide excision repair is a DNA repair mechanism that plays a critical role in maintaining genomic integrity. It is responsible for removing a wide range of DNA lesions, particularly bulky DNA lesions. The primary endogenous source of DNA damage is ROS, which result in oxidation of DNA bases, apurinic/apyrimidinic (AP) sites, and SSBs. Oxidative damage results in chemical modification to the ring atoms of purines, with the formation of 8-oxoG being the most common modification [110]. 8-oxoG is very mutagenic due to the propensity of DNA polymerase to insert adenine opposite to 8-oxoG. Such lesions are primarily repaired by base excision repair. Since ROS are the primary inducers of DNA damage in the context of NETosis and there is active oxidative DNA damage to NETs, we will focus on base excision repair [111].

### 4.1. Base Excision Repair Mechanism

Base excision repair is a critical mechanism for correcting oxidative DNA damage. The key steps of base excision repair are base removal, incision, end processing, repair synthesis, and ligation [110]. The process begins with base removal, where DNA glycosylases identify and excise damaged or inappropriate bases, leaving behind an abasic site. Next, incision occurs, as an apurinic/apyrimidinic (AP) endonuclease cuts the DNA backbone near the abasic site. Following this, end processing prepares the DNA for repair by removing any remaining sugar–phosphate residues and making the ends ready for new nucleotide insertion. During repair synthesis, DNA polymerase fills in the gap with the correct nucleotide, restoring the DNA sequence. Finally, ligation is carried out by DNA ligase, which seals the new segment into the existing DNA strand, ensuring that the repair is complete and that the integrity of the DNA is maintained.

#### 4.1.1. Base Removal

Damaged bases are removed from a DNA strand by specialized enzymes known as DNA glycosylases. These enzymes specifically target and recognize a variety of modified or damaged bases, excising them and leaving behind an abasic site, also referred to as an AP (apurinic/apyrimidinic) site. To date, several different DNA glycosylases have been identified, each responsible for addressing distinct types of base damage [110].

8-oxoG base-paired to cytosine is removed by 8-oxoguanine DNA glycosylase (OGG1), DNA glycosylase 1 (NEIL1), and nei-like DNA glycosylase 2 (NEIL2). 8-oxoG base-paired to adenine is removed by mutY DNA glycosylase (MUTYH). 8-oxoG can be further oxidized to give guanidinohydantoin (Gh) and spiroiminodihydantoin (Sp) [112,113]. Gh and Sp are removed by NEIL1, NEIL2, and NEIL3 [114]. Other products of oxidized bases that are removed by NEIL3 are 5-hydroxy-2′-deoxycytidine (5-OHC) and 5-hydroxy-2′-deoxyuridine (5-OHU) [114].

Formamidopyrimidine (Fapy) lesions are products of oxidative stress on purines [115]. Fapy base-paired to cytosine is removed by OGG1 and NEIL1 [116,117,118]. 4,6-diamino-5-formamidopyrimidine (FapyA) is removed by NEIL1 and NEIL2 [117]. 2,6-diamino-4-oxo-5-formamidopyrimidine (FapyG) is removed by nth-like DNA glycosylase 1 (NTHL1), NEIL1, NEIL2, and nei-like DNA glycosylase 3 (NEIL3) [114,118].

Uracil in DNA is the result of cytosine deamination. Several DNA glycosylases are responsible for removing uracil bases. Uracil base-paired to adenine is removed by uracil-DNA glycosylase 2 (UNG2), selective monofunctional uracil-DNA glycosylase 1 (SMUG1), and uracil-DNA glycosylase 1 (UNG1, in mitochondria). Uracil base-paired to guanine is removed by UNG2, thymine-DNA glycosylase (TDG), methyl-CpG binding domain 4 (MBD4), and UNG1 [119]. Ten-eleven translocation (TET) proteins also oxidise thymine to produce the product 5-hydroxymethyluracil (5-OHmeU) [120,121]. The modified base 5-OHmeU is removed by SMUG1, TDG, and MBD4.

Another common base modification is alkylation, usually in the form of methylation. 3-methyladenine, 7-methylguanine, and 3-methylguanine are removed by N-methylpurine DNA glycosylase (MPG), while 5-methylcytosine (5-meC) is removed by DEMETER (DME) [122]. 5-meC is oxidized by TET dioxygenases to form 5-formylcytosine (5-fC) and 5-carboxylcytosine (5-caC) [123]. These two modified bases are removed by TDG [123]. 5-hydroxymethylcytosine (5-OHmeC) is another product of 5-meC oxidation by TET and is removed by NTHL1 [124,125].

Modified bases can be the result of exogenous agents. 1,N 6-ethenoadenine is the product of a reaction between adenine with vinyl chloride or lipid peroxidation products [126]. Such lesions are removed by MPG [127]. 5-hydroxyuracil (5-OHU) is the product of ionization radiation [128]. The modified base 5-OHU is removed by SMUG1, NTHL1, NEIL1, and NEIL2 [128,129,130].

Wrongly paired bases are also repaired by base excision repair. Thymine base-paired to guanine (due to 5-mC conversion to thymine) is removed by MBD4 [131]. Oxidized adenine (2-OH-A) base-paired to cytosine or guanine is removed by MUTYH [132]. Hypoxanthine (Hx), the product of adenine deamination, is removed by MPG [133,134].

DNA glycosylases are divided into two groups: mono- and bi-functional glycosylases. The two types differ in their catalytic mechanism and ability to carry out AP lyase strand cleavage. In the case of mono-functional glycosylases, a water molecule is used as a nucleophile to attack the aromatic carbon on the base. This results in the base being released and the subsequent formation of an AP site [135]. In the case of a bi-functional glycosylase, an amine moiety is used as a nucleophile to attack the base. This results in a covalent Schiff base protein–DNA intermediate [136].

Bi-functional glycosylases exhibit endonuclease activity, being able to create a single-strand break. The cleavage takes place within the phosphodiester linkage 3′ to the AP site. This results in a 3′ end that is non-conventional and requires processing to allow for a polymerase to add nucleotides [137].

#### 4.1.2. Incision and End Processing

DNA glycosylases leave behind an AP site after removing the damaged base. An AP endonuclease then incises the DNA backbone 5′ to the AP site. This results in a strand break, with 3′-OH on one side and 5′-deoxyribose phosphate (dRP) on the other [137]. The most common AP endonuclease is APE1, and it is responsible for carrying out 95% of such incisions [138]. APE1 carries out a hydrolytic catalytic reaction which cleaves the phosphodiester bond. The reaction is helped by Mg^2+^, which stabilizes the negative charge of the phosphate oxygen atoms [139]. Certain residues on APE1 are required for its activity. In the case of APE1’s catalytic activity, it is dependent on Asp283 and His309 residues forming a triad structure with water [137]. Asp283, His309, Glu96, and Asp210 have also been reported to be necessary for APE1’s lyase activity [140,141]. APE1’s role in base excision repair extends beyond its endonuclease activity. APE1 can also serve to remove 3’blocked ends that result from bi-functional DNA glycosylase activity. APE1 can also carry out proofreading activity to remove mismatched nucleotides during the repair synthesis step of base excision repair [142]. Furthermore, the N-terminus of APE1 grants it redox regulation, which allows it to control gene expression though regulation of transcription factors (such as p53, AP-1, and HIF-1α) [143].

#### 4.1.3. Repair Synthesis

The removed nucleotide needs to be replaced by a DNA polymerase. A 3′-OH terminus and a template are needed for polymerase to carry out its function. Repair synthesis comes in two forms: short-patch and long-patch repair [144]. During short-patch repair, polymerase only adds one nucleotide. In the case of long-patch repair, between 2 and 12 nucleotides are added [137]. During long-patch repair, polymerase displaces nucleotides to make room for the newly synthesized strand, creating a flap intermediate. The displaced strand (or flap) gets cleaved by a nuclease (often flap endonuclease 1 (FEN1)) which leaves behind a ligation ready product [145]. Polymerase β (Polβ) is the primary polymerase that fills in short gaps, while Polδ (with the help of the DNA clamp proliferating cell nuclear antigen (PCNA)) fills in longer strands. However, Polβ has been found to also be able to carry out strand-displacement synthesis and, as a result, can carry out short-patch and long-patch base excision repair [146,147].

The core set of proteins that are required for short-patch base excision repair are DNA glycosylase, AP-endonuclease 1, DNA pol B, and DNA ligase I or III. PARP1 and XRCC1 have been reported to participate in base excision repair of some types of damage [148]. Other proteins play a role in damage signaling and chromatin remodeling. In the case of long-patch base excision repair, the core proteins are DNA glycosylase, AP- endonuclease 1, DNA pol delta/epsilon, PCNA, FEN1, and DNA ligase I [149].

The long-patch variant may be the dominant form during post-preparative base excision repair (initiated by UNG2 [150] or NEIL1 [151]); however, in most other situations, short-patch base excision repair dominates. The choice of pathway can, in some cases, depend on which glycosylases are activated [152]. Other factors found to play a role in influencing whether short-patch or long-patch base excision repair takes place include DNA damage type, protein interactions, and the differentiation state of the cell [153]. Another factor that has been found to help determine which form of base excision repair takes place is ATP level. It has been uncovered that in low-ATP settings, DNA ligation is not favored, which results in longer strands being synthesized. However, in high-ATP conditions, DNA ligation was found to be more likely to occur following the addition of a single nucleotide [146,154].

#### 4.1.4. Ligation

The last step of base excision repair is sealing the DNA break. This is carried out by DNA ligase. Using energy from phosphoanhydride hydrolysis, DNA ligase catalyzes the creation of a phosphodiester bond between the upstream 3′-OH end and the downstream 5′-PO_4_ end. The process is ATP-dependent. DNA ligase I is reported to be primarily involved in long-patch base excision repair, while DNA Ligase III (dependent on the scaffold protein XRCC1) is primarily involved in short-patch base excision repair [155,156]. While DNA ligase’s involvement in the final step of base excision repair is well known, it also performs a crucial function in the assembly of the initial repair machinery [157].

### 4.2. Nucleotide Excision Repair

Nucleotide excision repair is a versatile and crucial DNA repair mechanism that primarily addresses bulky DNA lesions and helix-distorting damage [158]. It repairs a variety of DNA injuries, including those induced by ultraviolet (UV) radiation, such as cyclobutane pyrimidine dimers (CPDs) and 6-4 photoproducts (6-4 PPs), which involve abnormal covalent bonds between adjacent pyrimidine bases [159]. Additionally, nucleotide excision repair repairs chemical-induced damage, including bulky adducts formed by polycyclic aromatic hydrocarbons (PAHs) found in tobacco smoke and environmental pollution, as well as aflatoxins, which are toxins produced by certain fungi [160,161]. By efficiently removing these lesions, nucleotide excision repair maintains genomic stability and prevents mutations that could lead to various diseases, including cancer. While nucleotide excision repair can occasionally repair certain types of oxidative damage, especially if the lesions cause significant distortion to the DNA helix, it is not the primary pathway for repairing oxidative DNA damage [162].

Nucleotide excision repair removes a wide range of DNA lesions, particularly those induced by ultraviolet (UV) light. The process begins with damage recognition, which occurs via two sub-pathways: global genome nucleotide excision repair (GG-nucleotide excision repair) and transcription-coupled nucleotide excision repair (TC-nucleotide excision repair) [163]. In GG-nucleotide excision repair, the Xeroderma Pigmentosum, Complementation Group C (XPC)–RAD23 Homolog B (RAD23B) complex initially recognizes DNA damage, followed by the recruitment of Xeroderma Pigmentosum, Complementation Group A (XPA) and Replication Protein A (RPA) to verify the lesion [164]. In TC-nucleotide excision repair, RNA polymerase stalls at the site of damage during transcription, prompting recognition by Cockayne Syndrome B (CSB) and Cockayne Syndrome A (CSA) proteins, which then recruit additional nucleotide excision repair factors [165,166].

Once the damage is recognized, the Transcription Factor II Human (TFIIH) complex, containing the helicases Xeroderma Pigmentosum, Complementation Group B (XPB) and Xeroderma Pigmentosum, Complementation Group D (XPD), unwinds the DNA around the damage site [167]. XPA and RPA scaffold and organize the damaged DNA and nucleotide excision repair enzymes [168]. Two endonucleases, Xeroderma Pigmentosum, Complementation Group G (XPG) and Xeroderma Pigmentosum, Complementation Group F-Excision Repair Cross-Complementation Group 1 (XPF-ERCC1), then make precise incisions on either side of the lesion [169]. Specifically, XPG cuts on the 3′ side of the damage and XPF-ERCC1 cuts on the 5′ side, excising an oligonucleotide fragment approximately 24–32 nucleotides long [170].

Following the removal of the damaged DNA segment, DNA polymerase δ or ε, aided by PCNA and RPA, fills in the gap using the undamaged strand as a template [171]. The final step involves DNA ligase I or DNA ligase III, which seals the newly synthesized DNA to the existing strand, thus completing the repair process [172]. This highly coordinated and efficient pathway ensures the maintenance of genomic stability by accurately repairing DNA lesions that could otherwise lead to mutations and various diseases.

### 4.3. DNA Repair in Neutrophils

Although neutrophils are terminally differentiated cells with a limited lifespan and do not actively transcribe or replicate their genomes, they still possess pre-made DNA repair proteins [173,174,175,176]. This capacity for DNA repair, while not as extensive as that seen in other immune cells like macrophages, is nonetheless crucial given their role in the immune response. Neutrophils, despite their short lifespan, have evolved mechanisms to address DNA damage, raising intriguing questions about the function and significance of their DNA repair machinery [48]. Research has shown that NETosis agonists can trigger widespread transcriptional activity throughout the genome [63]. This phenomenon may occur because the transcription machinery stalls at sites of DNA damage, which then activates DNA repair processes [177].

Neutrophils are equipped with a variety of DNA repair proteins, including OGG1 (8-oxoguanine DNA glycosylase 1), PCNA (proliferating cell nuclear antigen), PARP (poly(ADP-ribose) polymerase), and DNA polymerase β (DNA pol β) [173,174,175,176]. These proteins play critical roles in maintaining DNA integrity and managing oxidative damage. For example, PCNA, a DNA helix-clamping protein, is present in the cytoplasm of resting neutrophils [174]. Although PCNA is well-known for its role in DNA replication and repair in proliferating cells, its presence in neutrophils has been reported to also play a role in regulating apoptosis [174].

DNA damage signaling resulting from ROS production in neutrophils has previously been documented. One study reported that under certain conditions, such as a pro-inflammatory environment, neutrophils can produce large amounts of ROS. This excessive ROS generation can activate DNA damage signaling, leading to the suppression of cytokines and the induction of apoptosis [178,179].

## 5. DNA Repair Initiated by Oxidative DNA Damage Drives UVC-Induced NOX-Independent NETosis

ROS can be generated in neutrophils either through mitochondria or NOX. In the case of UVC-induced NETosis, the ROS are primarily derived from mitochondria. UV radiation is known to alter enzymes, leading to increased ROS levels [79]. However, the exact mechanism by which mitochondrial ROS regulate UV-mediated NETosis remains unclear. ROS serve various cellular functions, including the oxidation of macromolecules such as DNA, which has been extensively studied [180]. Cells have well-established DNA repair mechanisms to address oxidative DNA damage, primarily through the base excision repair pathway [153]. Despite the presence of various DNA repair proteins in neutrophils, their role in NETosis is not well understood. Therefore, a study was conducted to investigate the potential involvement of DNA repair machinery in the process of NETosis [181].

First, oxidative DNA damage was investigated to confirm that neutrophil DNA was indeed being oxidatively damaged by the large amounts of ROS produced during NETosis. It was uncovered that the NET DNA was extensively damaged by lesions. The presence of 8-oxoguanine, a primary marker of oxidative DNA damage, was found to be prevalent throughout the NET DNA. This confirmed that DNA was being oxidatively damaged by ROS during NETosis. This oxidative stress and the resultant DNA lesions could trigger the recruitment and activation of DNA repair machinery, highlighting a crucial aspect of the cellular response during NETosis. Understanding this damage and the subsequent repair mechanisms provides deeper insights into the complex interplay between ROS, DNA integrity, and immune responses in neutrophils.

As previously mentioned, neutrophils contain DNA repair proteins such as OGG1, PCNA, PARP, and DNA pol β [173,174,175,176]. The primary method for addressing oxidative lesions involves base excision repair, which encompasses the removal of damaged bases, incision, processing of ends, repair synthesis, and ligation [110]. Initial studies focused on whether active DNA repair occurs during NETosis. This was investigated by detecting the presence of PCNA on NETs, given that PCNA trimer clamps around DNA and plays a vital role in holding DNA polymerase, thus playing a key role in the base excision repair process [182]. At the end of NETosis, PCNA was found uniformly distributed across NET DNA, indicating widespread DNA damage and repair throughout the genome. While previous studies have uncovered that stored PCNA plays a role in regulating apoptosis [174], these new findings propose an additional role for PCNA in facilitating NETosis in neutrophils. This discovery highlights the dual functionality of DNA repair proteins in neutrophils, suggesting that these cells are not only equipped to manage oxidative DNA damage but also use their repair machinery to support the formation of NETs.

Given that both oxidative DNA damage and active DNA repair were observed during NETosis, the next step was to determine if these cellular functions were merely bystanders, activated due to environmental changes within neutrophils, or if they played a direct role in NETosis. By targeting different components of the base excision repair machinery, it was discovered that UV-induced NETosis could be significantly reduced by inhibiting APE1, PARP, or DNA Ligase. The process of repairing oxidized bases involves the removal of the damaged bases by DNA glycosylases and the APE1-mediated cleaving of DNA at the abasic site [173,174,175,176]. Following these events, PARP binds to the ends of single-stranded DNA, catalyzing the synthesis of poly ADP-ribose at the damage sites. These early actions initiate the recruitment of additional repair machinery, including DNA ligases [183]. At the damaged sites, chromatin undergoes extensive decondensation and nicking during the assembly of the initial three enzymes (APE1, PARP, and DNA Ligase) [157]. Subsequently, PCNA forms a trimeric ring encircling the DNA, enabling repair DNA polymerases β and δ to interact with the PCNA clamp and other repair protein complexes, thus concluding the DNA repair process [110,183]. Therefore, the initial steps of the base excision repair pathway, crucial for nick formation, play a pivotal role in maintaining chromatin stability and are essential for the proper execution of NETosis. This indicates that DNA repair mechanisms are not just passive responses to DNA damage but actively contribute to the formation of neutrophil extracellular traps (NETs) during NETosis.

Curiously, APE1 is an enzyme with multifunctional properties. It possesses DNA endonuclease activity in its C-terminal, whereas its N-terminal has redox activity [184]. APE1 is also recognized for its role in activating various transcription factors, including p53, particularly under oxidative conditions [184]. This raises questions about whether reducing NETosis by administering APE1 inhibitors stems from inhibiting subsequent transcription factor activation; however, findings suggest otherwise. Inhibiting other early stages of base excision repair similarly reduced NETosis, indicating that the decrease is likely due to blocking of APE1’s endonuclease activity. Furthermore, CRT0044876, an APE1 inhibitor, targets the active site of APE1, impairing its 3′-phosphodiesterase and 3′-phosphatase activities [185]. Since APE1’s two terminals have distinct functions, CRT0044876 does not affect its redox activity. Using this inhibitor significantly reduced UV-induced NETosis.

Interestingly, the story is not so clear-cut. The investigation revealed that the effectiveness of inhibiting the DNA repair pathway to abolish NETosis significantly depends on the specific target selected. Blocking the early stages of base excision repair reduced NETosis, but blocking the final phases of base excision repair (involving PCNA interactions and DNA polymerases β and δ) failed to reduce UV-induced NETosis [111]. This is most likely because these proteins do not participate in chromatin unwinding and nicking [157]. Once ROS levels become significant, chromatin decondensation occurs through nicking and unwinding simultaneously at many damage sites. At this point, the role of PCNA and the repair synthesis steps in rewinding DNA at local damage sites becomes inconsequential. DNA ligase, although primarily functioning in the later phases of base excision repair by sealing DNA nicks post-repair, is necessary for assembling the initial repair machinery [157]. This explains why inhibiting DNA ligase reduced NETosis despite its main function taking place in the latter stage of repair.

### Summary

Extensive studies have uncovered that DNA repair machinery activity is indeed necessary for UV-induced NETosis. It was discovered that the ability of DNA repair machinery to unwind chromatin is a crucial factor in driving NETosis. It was previously discovered that kinase activation and transcription are involved in NETosis, including UV-induced NETosis [63,64]. Building on these findings and the latest discovery, one proposed coherent model is that during UV-induced NOX-independent NETosis, kinases activate transcription factors, which subsequently initiate transcription. This transcription process halts at sites of oxidative DNA damage, attracting DNA repair machinery [177,186]. The repair machinery operates at these sites to unwind chromatin and create DNA nicks, leading to the formation of temporary sites of unwound chromatin. As these sites accumulate globally, their compounded effect culminates in the complete unfolding of chromatin and the onset of NETosis.

The base excision repair machinery steps in to repair the DNA by unwinding the chromatin, excising the damaged base, and nicking the chromatin. These actions—DNA nicking and chromatin unwinding—are crucial for triggering UV-induced NETosis. This unique DNA repair process highlights the significant role of ROS in driving NETosis. The involvement of DNA repair pathways in NETosis not only underscores the complexity of the process but also reveals the intricate link between oxidative stress, DNA damage, and the immune response. This insight provides a deeper understanding of how neutrophils use their DNA repair machinery to facilitate the formation of NETs in response to UV-induced damage.

## 6. Both Base Excision Repair and Citrullinated Histone Contribute to Calcium Ionophore-Induced NOX-Independent NETosis

While UV-induced NETosis is a form of NOX-independent NETosis, it is distinct from many other forms of NOX-independent NETosis, such as those induced by certain bacteria, in that it does not induce citrullinated histones in vitro. Generally, non-infectious sterile injuries trigger NOX-independent NETosis and rely on mitochondrial ROS [187]. Previous studies have demonstrated that calcium ionophores can induce histone citrullination in neutrophils [64,65,76]. Douda et al. highlighted the significance of mitochondrial ROS in calcium ionophore-induced NOX-independent NETosis [65]. However, it is noteworthy that inhibiting ROS does not completely eliminate NETosis under these conditions.

When comparing UV-induced and calcium ionophore-induced NOX-independent NETosis, both rely on mitochondrial ROS for neutrophil DNA oxidation. This raises questions about whether DNA repair plays the same role or to the same extent in other forms of NOX-independent NETosis. Further research is needed to determine the extent to which DNA repair processes are involved in various forms of NOX-independent NETosis and to understand the specific contributions of mitochondrial ROS and other factors in these processes.

A series of experiments uncovered that DNA repair pathways are involved in various forms of NOX-independent NETosis, but the extent of base excision repair involvement differs among these processes. Inhibiting mitochondrial ROS and DNA nick formation extensively suppresses UV-induced NETosis, demonstrating a strong reliance on these mechanisms. However, the same approach only partially suppresses calcium ionophore-induced NETosis, indicating additional factors at play.

Given the added role that histone citrullination plays in calcium ionophore-induced NETosis, it can be reasonable to visualize that both base excision repair and histone citrullination contribute to this form of NOX-independent NETosis. Histone citrullination, which is absent in UV-induced NETosis, suggests a more complex interplay of cellular mechanisms in calcium ionophore-induced NETosis. This implies that while mitochondrial ROS and DNA repair are crucial, the unique involvement of histone modification also significantly impacts the NETosis process.

In addition to base excision repair of oxidative DNA damage and histone citrullination, various kinase cascades, such as mitogen-activated protein kinases (MAPKs), are activated during different types of NETosis [61,63,64,65]. These kinase cascades are instrumental in activating transcription factors and promoting their assembly on DNA promoters [63,188,189,190,191]. Specifically, MAPKs contribute to the regulation of transcriptional processes, which are crucial for NET formation. Blocking transcription with Actinomycin D is detrimental to NET formation, as it effectively prevents NETosis. However, mRNA translation does not significantly affect NETosis, underscoring the critical role of transcription in this process [63,188]. Actinomycin D inhibits both NOX-dependent NETosis, which is induced by stimuli like PMA and LPSs, and NOX-independent NETosis induced by calcium ionophores such as A23187 and ionomycin, as well as UV radiation [64,78,192]. This inhibition is indicative of the universal reliance on transcription for NET formation, regardless of the NETosis pathway involved.

Transcription-coupled DNA repair may also play a significant role in NETosis. This repair mechanism involves the stalling of transcription machinery at sites of DNA damage and the subsequent recruitment of repair proteins. This process can aid in effective chromatin decondensation at multiple damaged sites, facilitating the formation of NETs [186]. Histone citrullination, which preferentially occurs at promoter regions [193], further supports this process. The coordinated action of histone citrullination and transcription-coupled DNA repair at oxidized DNA bases can enhance chromatin decondensation. Therefore, the interplay between these mechanisms can expedite efficient NETosis and highlights the complexity of the underlying cellular processes involved.

### Summary

Recent findings reveal that both chromatin unwinding by DNA repair mechanisms and histone citrullination are integral to calcium ionophore-mediated NOX-independent NETosis. This highlights the multifaceted nature of NETosis, suggesting that neutrophils use ROS and the oxidative DNA damage repair pathway across various forms of NETosis. In particular, the process of chromatin unwinding is essential for facilitating NET formation by allowing access to damaged DNA for repair. Concurrently, histone citrullination, which is a modification of histone proteins, specifically contributes to certain forms of NETosis that involve elevated intracellular calcium levels in neutrophils.

This distinction underscores that while ROS and the associated DNA repair mechanisms are universally involved in NETosis, histone modifications play a more targeted role. In cases where intracellular calcium levels are heightened, such as calcium ionophore-induced NETosis, histone citrullination becomes a crucial factor. This modification enhances chromatin decondensation, thereby promoting NET formation. These insights suggest that the regulation of NETosis is not only dependent on oxidative damage and repair processes but also on specific histone modifications that are activated under particular cellular conditions. Consequently, the role of histone citrullination in calcium-mediated NETosis exemplifies the complex interplay between various molecular mechanisms in neutrophil function and NET formation.

## 7. Base Excision Repair Is Also a Key Driver in NOX-Dependent NETosis

As the name suggests, NOX-dependent NETosis relies primarily on ROS produced by the NADPH oxidase 2 (NOX2) enzyme rather than mitochondrial ROS. This distinction is critical because the source of ROS can influence the molecular pathways and cellular responses involved in NET formation [194]. While much is known about the importance of ROS in this process, an important and exciting knowledge gap that required addressing was whether DNA repair mechanisms are involved in NOX-dependent NETosis, similar to their role in NOX-independent NETosis.

Given that mitochondrial ROS and the oxidative DNA damage repair pathway are integral to certain forms of NOX-independent NETosis, it was crucial to investigate if these repair processes are also active during NOX-dependent NETosis. Understanding this would help elucidate whether the mechanisms of chromatin decondensation and the recruitment of repair machinery to damaged DNA sites are universal features of NETosis, regardless of the ROS source. The involvement of DNA repair mechanisms in NOX-dependent NETosis could reveal new layers of regulation and potential therapeutic targets, particularly in diseases where NETs play a pathogenic role, such as autoimmune disorders and chronic inflammatory conditions.

Neutrophils activate NOX and generate ROS in phagosomes to combat microbial infections. However, when exposed to high levels of bacteria (e.g., MOI > 5) or bacterial components like LPSs, neutrophils produce excessive ROS leading to the formation of NETs [59,65]. Upon activation, NOX generates O_2_^−^, which is then converted to H_2_O_2_ by superoxide dismutase. Subsequently, myeloperoxidase catalyzes H_2_O_2_ to HOCl, and it can also convert to OH radicals [195]. These ROS oxidize macromolecules, including DNA [196]. This proves to be the case during NOX-dependent NETosis as well. A study uncovered that significant oxidative damage, specifically in the form of 8-oxoG, is present on NETs induced by both PMA and LPSs [111]. 8-oxoG is a common marker of oxidative DNA damage, resulting from the direct modification of guanine by ROS. The detection of this lesion on NETs suggests that the DNA released during NOX-dependent NETosis undergoes substantial oxidative modifications similar to those observed in NOX-independent NETosis.

The presence of 8-oxoG highlights that, despite the differences in ROS sources between NOX-dependent and NOX-independent NETosis, oxidative damage to DNA is a consistent feature across both pathways. This finding underscores the universal role of ROS in driving DNA damage during NET formation, regardless of whether the ROS are derived from NOX2 activity or mitochondrial processes. Furthermore, it suggests that the DNA repair machinery, particularly those components involved in base excision repair, might be recruited to address oxidative damage even in NOX-dependent NETosis.

As previously mentioned, neutrophils are equipped with a suite of DNA repair proteins, including OGG1, PCNA, PARP, and DNA pol β [173,174,175,176]. These proteins are integral to the base excision repair pathway, which is primarily responsible for repairing oxidative DNA damage. To investigate the involvement of these proteins in NOX-dependent NETosis, researchers focused on the localization of PCNA, a key player in DNA repair processes. PCNA acts as a sliding clamp, enhancing the efficiency of DNA polymerases during repair. The study revealed that PCNA was present on the DNA of NETs induced by NOX-dependent stimuli, such as PMA and LPSs. This localization is indicative of active DNA repair processes occurring during NETosis, as PCNA is typically recruited to sites of DNA damage to facilitate repair. The presence of PCNA on NET DNA suggests that oxidative damage prompts the activation of DNA repair mechanisms, specifically the base excision repair pathway. This pathway is crucial for recognizing and excising oxidized bases, such as 8-oxoG, and for initiating subsequent repair steps to restore DNA integrity.

Further investigations confirmed the importance of the base excision repair pathway in NOX-dependent NETosis. Inhibitors targeting key enzymes in this pathway, such as APE1 and DNA ligase, significantly reduced NET formation, underscoring the essential role of DNA repair in this context. These findings align with previous observations in NOX-independent NETosis, where base excision repair was also critical. The consistent involvement of the base excision repair pathway across different types of NETosis highlights a universal response to oxidative stress in neutrophils, irrespective of the ROS source.

Similar to what was observed in NOX-independent NETosis, the effectiveness of inhibiting the DNA repair pathway varies depending on the specific target chosen. Research revealed that blocking early pathway steps like APE1, PARP1, and DNA ligase suppresses NETosis induced by PMA, LPSs, *Pseudomonas aeruginosa*, and *Staphylococcus aureus*. However, inhibition of later factors like PCNA and DNA polymerases did not reduce NETosis. Blocking early steps appears to prevent the opening of chromatin that typically occurs when repair machinery gathers at sites of damage to nick DNA. Research supports our observations, showing that stages preceding DNA ligase recruitment do not require structural changes to nucleosomes [157]. When DNA ligase is inhibited, APE1 can act without altering chromatin structure, whereas blocking later steps allows extensive chromatin unwinding as more machinery forms [157].

## 8. Spontaneous NETosis in Untreated, Healthy Neutrophils Is Driven by Background ROS Production and DNA Repair

A unique form of NETosis occurs in a small percentage of healthy, untreated neutrophils, known as spontaneous NETosis. Unlike other forms of NETosis, which are typically induced by external stimuli such as pathogens or chemical agents, spontaneous NETosis arises without any overt activation. Recent findings suggest that autophagy, a cellular process involved in degrading and recycling cellular components, plays a significant role in this type of NETosis [197]. This process is particularly intriguing because it appears to be more prevalent in neutrophils from individuals with certain disease states, such as diabetes [198].

The phenomenon of spontaneous NETosis raises interesting questions about the underlying mechanisms, particularly concerning the roles of ROS and DNA repair pathways. Neutrophils, even in their resting state, are known to produce a baseline level of ROS [199]. This basal ROS production is typically involved in cellular homeostasis and the regulation of various signaling pathways. However, in the context of spontaneous NETosis, it is hypothesized that ROS may exceed this baseline level, leading to oxidative stress and subsequent DNA damage.

In the case of spontaneous NETosis, the involvement of ROS and DNA repair is not yet fully elucidated. However, considering the similar triggers and responses observed in other forms of NETosis, it is plausible that ROS-induced oxidative stress may also play a pivotal role here. The increased frequency of spontaneous NETosis in disease states like diabetes, which are characterized by chronic low-level inflammation and oxidative stress, further supports this hypothesis [200,201]. In these conditions, elevated ROS levels could exacerbate DNA damage, thereby increasing the likelihood of spontaneous NET formation.

Investigations have revealed that ROS and DNA repair mechanisms do indeed drive spontaneous NETosis in healthy neutrophils. However, the impact of inhibiting late-stage base excision repair differs from the effects observed in NOX-dependent and NOX-independent NETosis, where such inhibition typically does not affect ultimate NETosis levels.

In experiments, untreated healthy neutrophils exhibited background, or spontaneous, NETosis, which was effectively suppressed using a NOX inhibitor (DPI) or an ROS scavenger (N-acetylcysteine, NAC). This finding suggests that endogenously produced ROS significantly contribute to spontaneous NETosis.

Interestingly, when inhibitors targeting base excision repair steps that occur after chromatin unwinding were applied, an unexpected effect was observed. Specifically, the use of PCNA and polymerase β/δ inhibitors led to an increase in NETosis levels. Notably, blocking the activity of one of the polymerases resulted in only a partial increase in NETosis, whereas interfering with the interaction between PCNA and the polymerases caused a significant elevation in NETosis levels. In contrast, inhibitors targeting earlier steps in the base excision repair pathway resulted in a significant decrease in NETosis.

These outcomes suggest a complex role for DNA repair proteins in regulating NETosis dynamics. The involvement of polymerase β and polymerase δ in repairing DNA damage caused by endogenous ROS appears to be a crucial factor. Their activity helps to manage DNA damage while preventing excessive chromatin decondensation that could otherwise lead to increased NETosis, as was observed when the polymerases’ activity was inhibited. This suggests that inhibiting steps following chromatin unwinding could potentially lead to permanently unwound chromatin at damaged sites, where the damage remains unrepaired. This accumulation of locally unwound chromatin sites may aggregate and exert a global effect, potentially driving the chromatin unwinding process crucial for NETosis in neutrophils.

The suppression of spontaneous NETosis by targeting early steps in the base excision repair pathway indicates that these initial repair processes are vital for maintaining genomic stability and preventing uncontrolled NET formation. Inhibitors targeting early stages of DNA repair decrease NETosis by preventing the formation of sites with transient and persistent chromatin unwinding, acting prior to the unwinding phase [157]. This is supported by previous findings that demonstrate the DNA repair machinery’s ability to unwind chromatin [202].

### Summary

A key takeaway from these studies is that the effects of inhibiting DNA repair on NETosis levels vary significantly depending on the context. In activated neutrophils exposed to UV, PMA, LPSs, bacteria and calcium ionophores, inhibitors targeting PCNA and DNA polymerases showed no significant change in ultimate NETosis levels [111]. However, in neutrophils not subjected to external activation, inhibiting PCNA and polymerases notably heightened spontaneous NETosis. This finding can be explained by the notion that, in untreated neutrophils, the rates of oxidative DNA damage are relatively low. As a result, the DNA repair process can be completed efficiently by the available DNA polymerase and PCNA in most—but not all—neutrophils. This completion allows the repair machinery to disengage and the chromatin to rewind. Therefore, when PCNA and polymerases are inhibited, this may lead to the accumulation of unwound chromatin due to the failure to repair the nick created by APE1 at the DNA damage site. This accumulation could then trigger or exacerbate spontaneous NETosis, highlighting the critical role of these repair processes in maintaining chromatin stability and regulating NETosis dynamics.

On the other hand, in activated NETosis, the rapid accumulation of oxidative damage overwhelms the capacity of PCNA and DNA polymerases to repair all the damaged sites. This scenario results in the enzymes being unable to effectively address every site of DNA damage. Consequently, even when inhibitors target PCNA and polymerases, these interventions do not significantly alter the outcome, and NETosis continues unabated in activated neutrophils. This is because the volume and speed of damage exceed the repair capabilities of the available machinery, making it unfeasible for these enzymes to manage all repair sites effectively. This finding underscores the critical difference in the role of DNA repair machinery between spontaneous and activated NETosis and highlights how the cellular context and extent of DNA damage influence the outcomes of DNA repair inhibition.

These investigations highlight the contextual influence on neutrophil NETosis [203,204,205]. In environments rich in antioxidants, such as serum-containing blood with albumin, NETosis was observed to decrease. Conversely, in environments with limited antioxidants, such as tissue settings, neutrophils exhibited heightened susceptibility to NETosis. Our findings further reveal that in environments low in antioxidants (e.g., media lacking FBS, akin to extravascular spaces), inhibitors of PCNA and polymerases induce NETosis. In contrast, in high-antioxidant environments (e.g., media with FBS, resembling intravascular blood), neutrophils do not undergo NETosis when treated with PCNA and polymerase inhibitors. Thus, NETosis regulation exhibits context-dependent variability, with pathways responding differently to environmental cues.

## 9. Clinical and Technical Relevance

The universal role of DNA repair pathways in ROS-mediated NETosis carries significant implications for understanding the pathogenesis of various diseases, especially those involving inflammation and autoimmune disorders. As exposure to UV radiation increases and the prevalence of autoimmune diseases like lupus rises, it becomes crucial to delve deeper into the mechanisms behind NETosis [206]. The skin, being particularly susceptible to UV radiation [207], experiences DNA damage and subsequent perivascular inflammation [207,208]. This phenomenon is not only a potential driver of autoimmune responses but also a critical area of study for revealing novel mutations that could influence susceptibility to autoimmune diseases. Such insights could open new avenues for therapeutic interventions.

The importance of studying UV-induced NETosis extends beyond understanding disease mechanisms. It also has practical implications for treatment strategies. For instance, peripheral UV exposure is a common therapeutic approach for managing autoimmune skin diseases like psoriasis and vitiligo [209,210,211]. These treatments, while effective in reducing symptoms, also raise questions about the molecular pathways they influence. Specifically, the induction of NETosis or apoNETosis through UV exposure may play a role in the therapeutic outcomes or potential side effects. Understanding the molecular steps involved in these processes could help refine treatment protocols, potentially leading to more targeted and less harmful therapies.

The potential for therapeutic targeting of these pathways is substantial. By modulating DNA repair mechanisms or ROS levels, it may be possible to influence NETosis and its associated inflammatory responses. For example, in acute emergencies such as sepsis, targeting specific molecular pathways can have significant therapeutic potential. One such target is the base excision repair protein APE1. By inhibiting APE1 using pharmaceutical drugs, it may be possible to reduce the formation of NETs in the patient. NETs are known to contribute to the exacerbation of sepsis symptoms by causing additional inflammation and tissue damage [212,213,214]. Therefore, APE1 inhibitors could help mitigate the severity of sepsis by decreasing NET formation, thereby potentially alleviating some of the symptoms associated with this critical condition. This approach underscores the potential for targeted therapies to provide symptomatic relief and improve patient outcomes in acute inflammatory diseases.

Such strategies could offer new ways to manage autoimmune diseases, reducing the burden of chronic inflammation and preventing tissue damage. Additionally, understanding the role of UV-induced NETosis could help in developing protective measures for individuals at risk of excessive UV exposure, such as those with outdoor occupations or specific genetic susceptibilities.

Additionally, these studies’ findings could hold substantial implications for diseases and single-nucleotide polymorphisms (SNPs) associated with reduced activity or expression of DNA repair proteins. Mutations in genes responsible for DNA repair may contribute to inflammatory and autoimmune conditions like rheumatoid arthritis and lupus. For example, studies on a *POLB* mouse model with decreased efficiency suggest a link to lupus symptoms, potentially due to heightened NETosis resulting from the *PolB* mutation [74,215,216,217], along with other variations in repair proteins and enzymes [218].

The role of DNA repair in NETosis seems to be less about the actual repair process and more about the chromatin unwinding and nicking capabilities of the repair machinery. The term “futile” aptly describes this phenomenon, as the repair itself does not appear to be the critical outcome of the DNA repair activity in this context. Interestingly, this is not the first time a DNA repair pathway has been observed to play a role beyond its traditional function. A similar situation is observed with the mismatch repair pathway, a crucial system responsible for maintaining genomic stability by correcting base–base mismatches and insertion/deletion mispairs that occur during DNA replication and recombination [219].

In mismatch repair, a type of repair called “futile DNA repair” has been identified. This process involves the activation of signaling pathways that lead to cell cycle arrest and apoptosis rather than completion of the actual repair of DNA lesions [219]. This mechanism plays a significant role in maintaining cellular health by eliminating cells with potentially harmful mutations. However, in certain cancers, the inhibition of mismatch repair can lead to decreased apoptosis and increased cell survival, contributing to resistance against chemotherapeutic agents [220]. This resistance arises because the cancer cells, which would normally undergo apoptosis due to unrepairable DNA damage, survive and continue to proliferate.

This concept of “futile repair” parallels what has been observed in the base excision repair pathway’s involvement in NETosis. Inhibiting base excision repair, which is responsible for addressing oxidative DNA damage, can actually prevent NETosis from occurring in neutrophils. This suggests that the critical aspect of DNA repair in this context is not the completion of repair but rather the initiation of chromatin unwinding and DNA nicking.

The comparison between mismatch repair and base excision repair in these contexts highlights a fascinating aspect of DNA repair pathways: their roles are not limited to maintaining genomic integrity but can extend to other cellular functions, such as regulating cell death and immune responses. The concept of “futile repair” in NETosis underscores the idea that the mere engagement of DNA repair machinery can have significant biological consequences, even if the repair itself does not proceed to completion.

The potential therapeutic implications of these findings are both exciting and promising. An intriguing advancement in this area was achieved by a research group using nuclear-penetrating anti-DNA autoantibodies to inhibit NET formation. Specifically, the 3E10 nuclear-penetrating lupus autoantibody has been previously reported to localize to DNA in damaged tissues and inhibit DNA repair. This property was leveraged in cancer therapy, where it rendered cancer cells more susceptible to DNA-damaging agents by inhibiting their DNA repair mechanisms [221]. To reduce potential off-target effects, a modified version of this antibody, with the Fc region removed, was developed and named Deoxymab-1 (DX1) [222]. DX1 is currently under investigation as a potential treatment against tumors.

Interestingly, DX1 was also tested in differentiated PLB-985 cells treated with PMA, as well as in mouse neutrophils stimulated with PMA or ionomycin. In all these treatment conditions, the use of DX1 resulted in a significant decrease in NETosis, aligning with previous reports that demonstrated the role of DNA repair inhibition in reducing NET formation [222].

Another fascinating discovery was that DX1 did not affect histone citrullination levels [222]. This observation is consistent with the understanding that DNA repair mechanisms are involved in NETosis pathways both with and without histone citrullination. The distinct roles of DNA repair and histone modifications in the regulation of NETosis suggest that therapeutic strategies can be specifically tailored to target the DNA repair pathway without interfering with histone citrullination, thus providing a more nuanced approach to managing diseases characterized by excessive NET formation.

Autophagy has been reported as an important factor in the regulation of spontaneous NETosis, particularly in patients with diabetes [223]. This phenomenon is intriguing because spontaneous NETosis in diabetes patients is associated with increased levels of ROS, similar to observations made in healthy, untreated neutrophils [199]. The interplay between autophagy and DNA repair mechanisms is a fascinating area of study. It has been suggested that autophagy, a cellular degradation and recycling process, can be activated by genomic stress or damage. In this context, autophagy may play a protective role by promoting DNA repair pathways, thus helping to maintain cellular integrity and prevent further damage [224].

This connection between autophagy and DNA repair is particularly significant in the context of NETosis, as both processes can influence chromatin dynamics. The hypothesis that autophagy could lead to chromatin decondensation via the production of ROS and the activation of DNA damage repair pathways is a compelling area for further investigation. Additionally, the relationship between autophagy and NETosis could have significant implications for understanding the pathology of diseases such as diabetes, where oxidative stress and autophagy dysfunction are common. In diabetic patients, heightened levels of ROS and chronic inflammation are prevalent, and these factors can exacerbate the rate of NETosis.

Another study revealed that phosphorylation of the Ser-139 residue on histone H2AX, a marker for double-strand breaks [225], occurs during NETosis [226]. This discovery is particularly intriguing, as it suggests that the double-strand break repair pathway may play a role in the NETosis process. This opens a compelling avenue for future research, focusing on the involvement of double-strand break repair in NET formation and the potential therapeutic targets within this pathway. The study also observed an upregulation of the chemokine receptor CXCR4 in NETotic neutrophils, which is notable because CXCR4 is associated with cellular responses to stress and damage repair mechanisms [227]. Interestingly, the application of hydrogen (H_2_) was found to significantly reduce NET formation and decrease the population of CXCR4-expressing cells. This implies that H_2_ might inhibit NETosis through a mechanism that involves mitigating DNA damage [226]. The reduction in CXCR4-expressing cells further supports the notion that H_2_ potentially alleviates the DNA damage response, thereby reducing NETosis.

The involvement of H_2_ in reducing NETosis highlights an important aspect of the process—managing oxidative stress and its consequent DNA damage. The findings suggest that therapies aimed at reducing DNA damage or enhancing repair mechanisms could be beneficial in controlling NETosis, especially in conditions where excessive NET formation leads to tissue damage and inflammation.

Since DNA repair actively occurs during NETosis, it is intriguing to consider whether nucleotide synthesis also takes place in neutrophils. This area of research is relatively nascent, but early findings suggest a connection between metabolic pathways and the synthesis of nucleotides [228]. Notably, a compelling report highlighted the essential role of the pentose phosphate pathway (PPP) in NETosis [229]. The PPP is crucial because it provides NADPH, which is necessary for the NADPH oxidase (NOX) enzyme complex to produce superoxide radicals.

An interesting aspect of the PPP’s involvement in NETosis is the production of ribose-5-phosphate, a significant by-product of this pathway [230]. Ribose-5-phosphate is a vital precursor for the biosynthesis of nucleotides, the building blocks of DNA and RNA [231]. This raises the intriguing possibility that, in addition to providing reducing power for ROS generation, the PPP might also contribute to the replenishment of nucleotide pools in neutrophils during NETosis. The synthesis of new nucleotides would be crucial for supporting the extensive DNA repair activities observed during NET formation, particularly given the massive DNA damage and chromatin remodeling involved.

The potential link between the PPP and nucleotide synthesis in neutrophils could have significant implications for understanding the metabolic requirements of NETosis. It suggests that neutrophils may upregulate certain metabolic pathways not only to produce ROS but also to support DNA synthesis and repair. This dual role of the PPP in both energy metabolism and nucleotide biosynthesis could be a key factor in maintaining the delicate balance between DNA damage and repair during NETosis.

## 10. Conclusions and Future Directions

In conclusion, DNA repair mechanisms, particularly base excision repair, play a pivotal role in the process of NETosis across different contexts, including NOX-dependent and NOX-independent pathways. The evidence shows that both polymerase β and polymerase δ are involved in the repair processes during NETosis, in line with recent research blurring the traditional distinctions between long-patch and short-patch base excision repair. This dual involvement suggests a more complex interaction of DNA repair pathways in neutrophils than previously understood. The activation of these pathways not only facilitates the unwinding of chromatin, which is crucial for the formation of NETs, but also highlights the unique role of DNA repair in the immune response of neutrophils beyond simply repairing damage.

It is also important to note that DNA replication, a process typically associated with PCNA and polymerase δ, is not necessary for NETosis [232]. This observation further supports the notion that the DNA repair mechanisms, rather than DNA replication, are the critical driving force behind NETosis. The involvement of DNA repair machinery, particularly base excision repair, highlights the unique adaptations of neutrophils to handle oxidative DNA damage and facilitate NETosis. Inhibition studies, particularly with PCNA and polymerases, further emphasize that while DNA repair processes are vital in regulating NETosis, the specific roles of different repair pathways and enzymes may vary depending on the oxidative stress levels and the nature of the stimuli. Further research is needed to fully elucidate the specific roles and mechanisms of these DNA repair pathways during this unique form of cell death.

It is crucial to recognize that the studies have been conducted in controlled laboratory settings, often using chemical inhibitors and genetic knockdowns in isolated human/mouse cells or differentiated cell lines. These experimental conditions provide valuable insights but do not fully replicate the complexities of live organisms. In vivo, the environment is far more heterogeneous, with numerous interacting factors that can influence outcomes in unpredictable ways. The presence of diverse variables and their interactions in live settings adds a significant layer of complexity to our understanding of NETosis and its consequences. To fully appreciate the implications of recent discoveries, it is essential to conduct studies in more biologically relevant and complex environments. This approach will help to better gauge and understand the real-world ramifications of DNA repair in NETosis, particularly in the context of human health and disease.

Future directions for research include a deeper exploration of the specific molecular mechanisms by which polymerase β and polymerase δ contribute to base excision repair during NETosis. Investigating the exact nature of chromatin modifications and the role of histone citrullination alongside DNA repair processes will be critical. Additionally, understanding the regulatory mechanisms that determine the shift between long-patch and short-patch base excision repair in neutrophils could provide insights into the cell-specific adaptations of DNA repair pathways. Traditionally, long-patch base excision repair has been associated with PCNA and polymerase δ, while polymerase β has been linked with short-patch base excision repair. This raises the question of which type of base excision repair predominates during NETosis. Recent research, however, has suggested that PCNA and polymerase δ can also participate in short-patch base excision repair, while polymerase β can be involved in long-patch base excision repair [233,234,235]. This finding complicates the classical understanding and suggests that both polymerases may play roles in NETosis that differ from their traditional assignments.

Long-patch base excision repair is generally considered less critical in terminally differentiated cell types, like neutrophils, compared to short-patch base excision repair [236]. This suggests that short-patch base excision repair is primarily responsible for facilitating chromatin unwinding during NETosis. However, the exact contributions of each base excision repair pathway in this context require further investigation to be clearly delineated and understood.

ROS are known to cause various types of DNA damage, including the formation of large adducts on DNA. These adducts, which result from covalent bonding between DNA and reactive molecules, are typically bulky and can distort the DNA helix. Such damage cannot be effectively repaired by the base excision repair pathway, which primarily deals with small, non-bulky lesions like single-base modifications. Instead, these large adducts require the nucleotide excision repair pathway for proper repair [237]. Nucleotide excision repair is adept at recognizing and removing bulky lesions, making it an essential repair mechanism for maintaining genomic integrity in the presence of ROS-induced damage. Thus, the involvement of nucleotide excision repair in handling ROS-induced DNA adducts warrants further investigation to fully understand its role in NETosis and other oxidative stress-related processes.

In the context of UV-induced DNA damage, another critical lesion that must be considered is the formation of cyclobutane pyrimidine dimers (CPDs) and 6-4 pyrimidine-pyrimidone photoproducts ((6-4)PPs). These lesions are a significant consequence of UV exposure, typically involving thymine or cytosine bases in DNA. The formation process begins when UV radiation induces the covalent bonding of adjacent pyrimidine bases, leading to the creation of these photoproducts. This bonding occurs when UV light causes the electrons in the double bonds of these bases to become excited, resulting in the formation of a cyclobutane ring structure between two adjacent pyrimidine bases in the case of CPDs, or a single covalent bond in the case of 6-4 PPs [238]. Their repair is primarily facilitated by the nucleotide excision repair pathway [239]. Given the pivotal role of nucleotide excision repair in correcting UV-induced DNA damage, it represents another crucial area of study, especially in understanding the full spectrum of DNA repair mechanisms engaged during UV-induced NETosis.

**Figure 1:**
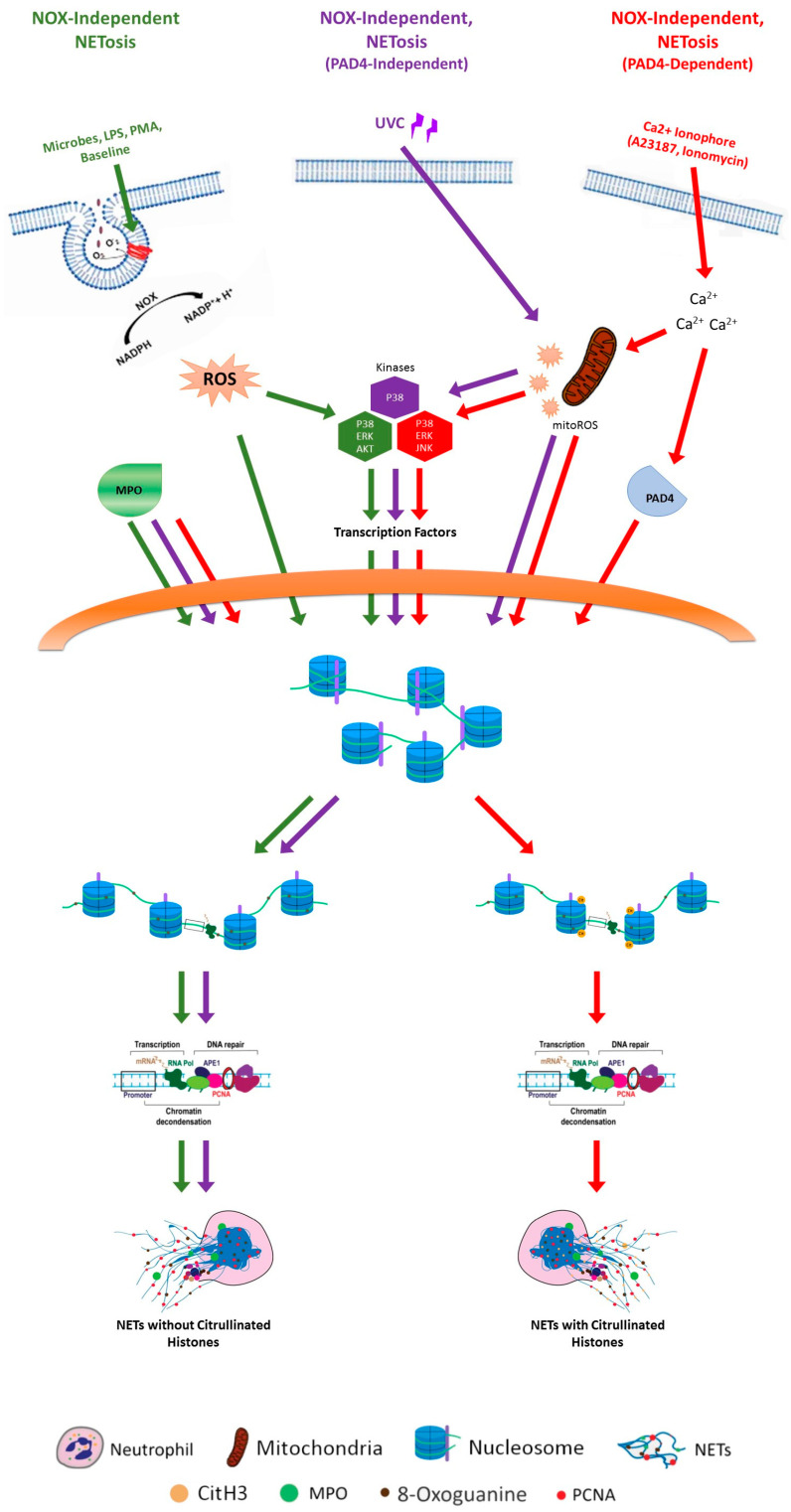
Summary figure detailing the mechanisms involved in NOX-dependent NETosis (green arrows) and NOX-independent NETosis with (red arrows) and without (purple arrows) peptidylarginine deiminase 4 (PAD4) activation. Following the initiation of NETosis, the production of ROS occurs, which can originate from either NOX or mitochondria. ROS induce oxidative DNA damage. Kinase activation, in turn, activates transcription factors. Transcription coupled with DNA repair is then initiated, leading to the unwinding and nicking of DNA. In the context of calcium ionophore-induced NETosis, PAD4 results in the citrullination of histones. Ultimately, these processes result in the release of DNA extracellularly as NETs.

Further studies should also explore the implications of these findings for diseases characterized by chronic inflammation and oxidative stress, where NETosis plays a significant role. These include conditions like diabetes, autoimmune diseases, and chronic infections, where understanding the regulation of NETosis could lead to new therapeutic strategies. Additionally, the potential for targeting specific DNA repair pathways in the modulation of NETosis offers a promising avenue for therapeutic intervention, particularly in diseases where NETosis contributes to acute pathogenesis, such as sepsis. Thus, the future research agenda includes both fundamental investigations into the molecular biology of NETosis and applied research aimed at developing clinical interventions (Figure 1).
